# No differences in executive functions between female and male young talented football players

**DOI:** 10.3389/fpsyg.2025.1635329

**Published:** 2025-08-19

**Authors:** Jennifer Lehmann, Stefanie Pietsch

**Affiliations:** Faculty of Human Science, University of Regensburg, Regensburg, Germany

**Keywords:** football, development, sex differences, executive functions, young

## Abstract

**Introduction:**

Research regarding the relationship between cognitive performance and sport performance in young talented football players has mainly been investigated in male athletes. Only few studies have addressed these aspects in female athletes. Thus, this quasi-experimental cross-sectional study aims to contribute to research addressing possible sex differences in the relationship of cognitive aspects in young talented football players during a selection assessment for a representative team and possible adjustments in this process or training processes in consideration of potential sex differences.

**Methods:**

Therefore, the participants were assessed using a general questionnaire, the Number Connection Test (ZVT), and a resilience questionnaire (1st session), as well as the 2-back test, the Flanker test, and the Switching task (computer-based, 2nd session). In total 59 male and 47 female athletes aged between 12 and 13 years were included.

**Results:**

The results of this study did not show any differences in executive function performance between female and male talented football players. Additionally, when examining potential differences between players selected for a representative team and those, who were not, no such differences were detected. Neither in general, nor sex-specific.

**Discussion:**

The results suggest that further research is necessary to clarify whether there are sex differences among talented young football players and whether these differences play a role in the selection for a representative team.

## Introduction

The role of cognition in sports, particularly executive functions in football^1^, remains a topic of ongoing debate. Other aspects are occasionally included as well, and in the present study, resilience is considered as a secondary aspect. While recent research, for instance Beavan et al. (2020) question the overall significance of executive functions in football, especially regarding talent scouting for youth elite football players, other studies emphasize the importance of cognitive functions in football (e.g., Habekost and Ovesen, 2024; Verburgh et al., 2014; Vestberg et al., 2012). While the study by Beavan et al. (2020), showed that executive functions predominantly develop between the ages of 10 and 15, but show no differences in development trajectories between football players and the general population, different meta-analyses generally support a connection between cognitive abilities and sports performance (Mann et al., 2007; Scharfen and Memmert, 2019; Voss et al., 2010), revealing small to medium effect sizes that suggest elite athletes possess superior cognitive skills compared to non-elite athletes (Formenti et al., 2022). This is further supported by the general cognitive model developed by Habekost and Ovesen (2024) in elite football players that links cognitive processes to specific playing situations, namely the three stages assessment of the current play situation, action section and execution and the stage assessment of outcome and feedback-based learning. Beavan et al. (2020) and others argue against a substantial link between cognitive performance and sports expertise (Kida et al., 2005; Lundgren et al., 2016). In team sports, cognition is often associated with quick situational assessment and the ability to adapt strategies, including inhibiting certain responses. These cognitive abilities fall under the category of executive functions, which begin to develop before early adolescence (Luciana et al., 2005). Executive functions include core skills such as working memory, inhibition, and cognitive flexibility (Diamond, 2013; Myiake et al., 2000). Working memory refers to the ability to retain and manipulate information mentally, while inhibition is the ability to control attention, behavior, and thoughts to override impulsive actions in favor of more appropriate responses (Diamond, 2013). Cognitive flexibility involves the ability to shift perspectives and adapt to changing demands (Diamond, 2013). In sports, executive functions are crucial for various aspects, including interaction with the match environment (Jacobsen and Matthaeus, 2014). Examples of how executive functions influence football include deviating from intended passing routes when these are blocked by opponents or when teammates are better positioned, as well as applying pre-rehearsed plays and adapting them to changing game situations.

While some studies have explored possible differences in cognitive functions between female and male athletes, others have not addressed potential sex variations. Vestberg et al. (2012) included both male and female football players in their measurement of general executive functions, revealing that high-division football players outperform their low-division counterparts, with both groups surpassing a norm group representing the general population. This suggests that cognitive functions are important for the success of football players, regardless of sex since no sex differences were found. However, Vestberg et al. (2017) extended these findings only to young elite male football players. The study showed that compared to non-elite players, male youth elite football players exhibited higher executive functions, which are deemed crucial for football success. Similarly, Verburgh et al. (2014) focused on male football players, reporting superior executive functions, in particular inhibition and on the Attention Network Test, among highly talented athletes compared to amateur football players. Additionally, (Huijgen et al. (2015) conducted a study comparing cognitive functions in male elite youth football players with sub-elite counterparts, favoring elite youth football players in inhibitory control. The correlational relationship of inhibitory control with physical abilities, for example sprint capacity, as well as tactical performance was also established in the study by (Carnevale et al. (2022). This is further supported by the study conducted by (Lovecchio et al. (2021) who investigated inhibitory control and agility in seven-year-old children playing football. Most of the variance in physical abilities and tactical performance was explained by inhibitory control, but they also found the involvement of cognitive flexibility in this construct. While the evaluated only male under 15 football players, female athletes were not addressed. This relationship is partly explained by the fact that better cognitive control processes are also reflected in faster motor responses (e.g., Chang et al., 2017; Lovecchio et al., 2021). Addressing the gap in female data regarding executive functions and football players, (Beaven et al. (2022) conducted a groundbreaking mixed-longitudinal study. They analyzed executive functions in elite female football players and found developmental patterns that are comparable to those observed in the general population regarding age effects. Another study that addresses executive functions in female football players is the one conducted by (Spielmann et al. (2023). They included, next to executive functions, personality aspects in the study, comparing them across the age range from 16 years to adults for both female and male athletes. While they did not focus primarily on sex differences, they reported different scores in neuroticism for male and female athletes. Yet, they did not find specific consistent influence of executive functions and personality traits but merely suggested that further research is needed to understand the complexity of possible relationships. This holds true for addressing sex differences in this research topic since, even when studies (Friedman et al., 2008; Huizinga et al., 2006) report no sex differences between normal populations and young sportive adults, there is still a lack of consistent results regarding the complex understanding of possible relationships between personality traits and executive functions.

Although, the previous mentioned studies are highly interesting as they collect essential data on female football athletes, it also highlights that there is still room for further research, as only a few studies have investigated female young talented football players and compared them to male counterparts. This is even more relevant in the context of whether executive functions are relevant in football.

While the significance of EF (executive functions) is still viewed controversially, the importance of cognitive abilities is emphasized in the scientific research addressing the relative age effect in the selection of young football players. (Beaven et al. (2022) addressed data of female athletes already playing in a specific club system. This study wants to assess data on a more comprehensive level, namely on a state level rather than within a club system. While in Germany young football players are either already playing in the youth academy of elite football clubs, there are also local youth performance center. These centers are part of the regional state level football organization and here players are also selected regarding their talent to play in selected teams on a state level rather than already playing in clubs. A selection of these players is carried out within the process for the next year's cohort. These players are then further supported in their development before possible changing to the youth academies of elite football clubs. This can be relevant in talent development and identification, which is why the focus at the state level should also be addressed, as it is in this study. Up to date, no such study investigating possible executive function differences in talented football players exist and this study tries to establish if such differences exist and whether it is worth including those aspects inter alia in selection process. Furthermore, the question of how executive functions can be integrated into talent identification should also be addressed in this study. While (Sakamoto et al. (2018) have already used executive functions as a prognostic tool for talent identification, it remains unclear whether this integration is truly effective. As Scharfen and Memmert (2019) point out, other factors may also play a decisive role in this context and potentially outweigh the importance of executive functions. This indicates that further research is needed in this area. Although recent studies by Habekost and Ovesen (2024) and (Haugan et al. (2025) suggests that executive functions are highly relevant for game intelligence in elite football players, this aspect has not yet been sufficiently examined in youth development. Therefore, the present study aims to address this gap.

To draw possible comparable data, this study is guided by the study of Lehmann (2023) regarding the setting of the study, which evaluated executive functions as well as resilience in young elite male football players. Due to a comparable setup, the factor resilience will be gathered in this study as well. In this context, resilience is defined as an individual's ability to cope with negative experience in such a way that they demonstrate more positive outcomes (Parsons et al., 2016). Furthermore, resilience is closely linked to the ability to adapt to stressors (Masten, 2001), which, in the context of sports, can be both cognitive and psychological in nature. In sports generally, and particularly in football, resilience is often associated with experiencing lower levels of stress (Secades et al., 2017) or is considered a key factor in achieving high performance levels (Holt and Dunn, 2004). However, resilience is not only relevant in performance contexts; it has also been associated with greater success in talent identification and development (Sarmento et al., 2018). (Den Hartigh et al. (2024, p. 564) conceptualize resilience as “a dynamic process of bouncing back to normal functioning following stressors,” with stressors referring, for example, to various types of load—particularly physiological and cognitive load. The performance-impairing effect of such mental load has been demonstrated by (Gantois et al. (2019) and (Moreira et al. (2018), who found that players' performance deteriorated following mental fatigue induced by a Stroop task, which assesses components of executive functioning. In these cases, cognitive load directly affects athletic performance. A direct link between executive functions and resilience was also examined by Shariati and Nasiri (2024), who found that individuals with higher resilience scores also performed better in measures of inhibition and cognitive flexibility. However, it should be noted that this study did not include athletes. The connection between resilience and executive functions is often explained by the fact that executive functions are seen as a positive internal resource that supports adaptive responses under stress (Maley et al., 2016; Shields and Moons, 2017; Zhang et al., 2019). (Zhang et al. (2019) argue that executive functions contribute to goal-directed behavior and thereby strengthen individuals' sense of self-efficacy. This aligns with Masten's (2014) view that self-efficacy, as part of a broader motivational system, may represent a core component of resilience. In football specifically, this could imply that young players with more developed executive functions are better equipped to cope with stress during selection processes—and may even be able to convert such stress into positive psychological presence and performance on the field. The multifactorial significance of resilience in football is also emphasized by (Ashdown et al. (2025).

But how do different sexes cope with this stress, and what role does the protective factor of resilience play in this context? Hölling and Erhart (2007) showed in their survey that female participants aged between three and seventeen were more likely to display emotional problems, whereas male participants tended to show behavioral issues throughout this age span. These potential problems may influence resilience, which in turn could affect cognitive performance in both sexes—especially in females, due to self-critical thoughts and beliefs, which could ultimately impact performance in football. Therefore, this study aims to include the aspect of resilience in relation to the cognitive performance of both sexes.

This is also consistent with the fact that no study currently exists comparing resilience scores between young female and male soccer players.

The main focus of the present cross-sectional study is to investigate potential sex differences in executive functions between male and female young talented football players. Specifically, the study aims to examine whether executive functions are linked to the selection process for a representative team on a state level or not. Therefore, it is expected that selected players demonstrate superior executive functions compared to those not selected (Huijgen et al., 2015).

## Materials and methods

### Participants

Based on an a priori power analysis using G^*^Power for the MANOVA (Faul et al., 2009), a total sample size of 102 participants was calculated (1-ß = 0.8) to detect small to medium effect sizes (*f*^2^ = 0.08) at α = 0.05. This sample size is even larger than that used in various other studies conducted so far (e.g., Scharfen and Memmert, 2019, with 15 participants; Verburgh et al., 2014, with 84 participants; Vestberg et al., 2012, with 31 male and 26 female participants). This study involved 59 female and 47 male young talented football players from a German state level junior performance center. The participating players were aged between 12 and 13 years (female: *M* = 12.31, *SD* =.57; male: *M* = 12.62, *SD* = 0.49). All players primarily competed in football and played at a state level. Both female and male participants were tested while they underwent an assessment where their football performance was evaluated, and selections were made for a representative team. In this context, talented football players are understood to be those who stand out due to their current footballing skills and, in terms of development, have the potential to progress into national young teams for their age group and, in the long term, possibly into a professional football career. Therefore, they are selected due to their recognizable talent for the aforementioned process.

Participants were recruited through their federation coach 1 month prior to their football assessment in 2022 and 2023. Inclusion criteria for participants were parental consent, their own consent, and participation in the assessment. Exclusion criteria included the absence of a signed consent form from either the parents or the participant, as well as any psychological disorders or uncorrected visual disorders. Consent forms were missing for one girl and 13 boys, so they were not tested. No participants had to be excluded due to illness. All participating children, as well as their parents, provided written informed consent.

### Measures

The measurements were obtained from the study already published by Lehmann (2023). This refers exclusively to the methodological framework and does not involve the use of any pre-selected data.

#### Demographical questionnaire

In the self-generated demographical questionnaire, personal information was collected as well as details about participant‘s football involvement. This included inquiries about their football league affiliation, the extent of their commitment to sport measured in weekly hours, and the time dedicated to football training each week.

#### Processing speed

The evaluation of cognitive processing speed utilized the Number Connection Test (Oswald, 2016), comparable to the Trail Marking Test (Reitan, 1956). During this assessment, participants were directed to quickly link presented numbers in ascending order. The evaluation included four sheets of paper, along with two practice examples. The practice sheets featured numbers 1–20 in a random sequence, while the test sheets included numbers 1-90 in a random order. Administered as a group test, each sheet allowed a 30-s time limit. The resulting scores were then converted into scores using standardized test norms. The correlation between processing speed, assessed by the number connection test, and the outcomes of a standard IQ test ranges from *r* = 0.60 to 0.80 (Vernon, 1993). The internal consistency and reliability over a six-month period, determined through test-retest reliability, are approximately *r* = 0.90–0.95.

#### Executive functions

The essential cognitive processes of executive functions, including updating, inhibition, and cognitive flexibility, were assessed using the 2-back task, the flanker task, and the switching task. Each of these activities was performed using Open Sesame, an experimental software application, on a 15-inch laptop. The screen was positioned approximately 50 cm away from every participant.

##### 2-back task

Derived from the n-back task proposed by Kirchner (1958) to assess working memory and its capacity, the 2-back task is a specific variant known as “updating”. In this task, participant viewed a series of sequentially displayed letters and determined if the presented letter matched the one shown two positions earlier, responding with a right mouse-click. No response was required if the letters did not align. Each letter appeared for 500 milliseconds with the next letter emerging 2,500 milliseconds later, regardless of response. The assessment included a preliminary practice round with ten trials and feedback, followed by three main sets of experimental trials, totaling fifty each. Feedback was not provided during these experimental trials, which consisted of ten target items and forty distractors. Participant reaction times and accuracy for the target elements were meticulously recorded.

##### Flanker task

For the flanker task (Eriksen and Eriksen, 1974), employed to assess inhibition, more specifically perceptual inhibition, participants were presented with combinations of letters, with a central letter surrounded by three other letters on both sides. Participants pressed the left mouse key for a central H or K, and the right mouse key for an S or C. Surrounding letters could also be H, K, S or C, resulting in congruent and incongruent trials. The neutral condition involved the letters A or P. A total of 24 tasks (eight for each condition) and 96 trials were conducted. Each trial persisted until the participant responded, and a new trial emerged 500 ms after the response. Both reaction time and accuracy were logged. Participants underwent ten practice trials with feedback before experimental trials, which did not include feedback.

##### Switching task

**Figure 1:**
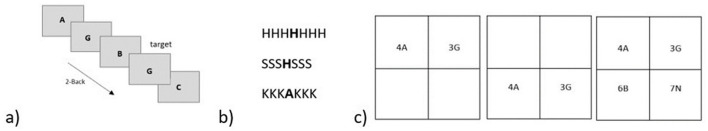
Examples for the executive function tasks. **(a)** presents the 2-back task, **(b)** represents 3 examples of a congruent, incongruent, and a neutral condition, **(c)** presents examples for the 3 blocks in the Task Switching task, where the number-letter combinations were not always presented in all fields, but only exemplarily shows in which fields the combinations may appear.

The Switching Task assessed cognitive flexibility using a modified version of the Number–letter task by Rogers and Monsell (1995), as outlined by (Myiake et al. (2000). Participants responded based in the location of a number-letter pair in four quadrants. In the first two quadrants, they determined if the number was odd or even; in the lower two quadrants, whether the letter was a consonant or a vowel. The task comprised three blocks. In the initial block, number–letter pairs were exclusively displayed in the upper quadrants for a total of 32 target trials. Subsequently, in the second block, the pairs were exclusively shown in the lower quadrants for another 32 target trials. The third block introduced a new challenge: number-letter pairs presented in a clockwise rotation across all four quadrants, requiring task shifts in half of the trials. Each block began with 12 practice trials with feedback. However, feedback was not provided during the actual main trail. Participants responded by pressing a button, with a 150 ms interval between response and stimuli. Shifting cost, measuring the impact of task-switching, was computed by comparing the average reaction times of the first two blocks with those of the third block, specifically for trials requiring a task shift. Examples of the three executive function tasks are shown in Figure 1.

#### Resilience

To assess an individual's capacity to manage psychosocial stress, the RS-11 Resilience Scale RS-11 (Schumacher et al., 2005), a condensed German adaption of the scale introduced by Wagnild and Young (1993), was utilized. During this procedure, participants were asked to evaluate eleven attributes using a seven-point-rating scale from 1 (I do not agree) to 7 (I completely agree). The rating results of the eleven attributes were then summed, forming an overall resilience score, which was used for further calculations. (Schumacher et al. (2005) validated this scale for people age 14 till 95 years of age.

### Experimental procedure

Quasi-experimental cross sectional design. All participants were tested during their football assessment, resulting in two separated test session, one for the female and one for the male assessment, due to different assessment dates. The test sessions themselves were conducted in the same way for both female and male participants. Due to the large number of participants, the group was divided into two equal-sized groups at the first testing time point, which were tested sequentially using the following test procedures for the paper-pencil tests: general questionnaire, resilience questionnaire, and processing speed. Those tests lasted approximately 25 min. On the following day, the entire group was divided into smaller groups of 10 participants, who were tested in consecutive sessions. On the second test day, the computer-based test procedures for executive functions were conducted. Executive functions were assessed individually on a computer screen, with the order of the tests randomized and counterbalanced for each participant. A study leader supervised, explained, and administered all testing sessions.

### Data analyses

Reaction time data (RT) was trimmed for outliers in all tests related to executive functions. Any RTs more than 2SDs below or above the mean for each condition and subject were excluded. Subsequently, multiple analysis of variance, MANOVAS, Bonferroni corrected, were conducted for the dependent variables of working memory and inhibition, shifting in both divisions. Due to the comparable setup of Lehmann (2023), resilience was included in the analysis as dependent variable as well. The independent factors were sex and team status (representative vs. non-representative team). To address multiple testing, the significance level was Bonferroni corrected. IBM SPSS Statistics V28.0 was utilized for data analysis, and the Shapiro Wilk test as normality check was conducted.

## Results

### Participants

Overall, female and male football players differed significantly regarding terms of age (*F*_(1, 103)_ = 8.52, *p* < 0.05), with male slightly older than females, and processing speed (*F*_(1, 103)_ = 17.94), *p* < 0.01), with faster processing speed for the female participants than the males, while no significant differences are found for frequency of training (*F*_(1, 103)_ = 0.12, *n.s*.) as well as hours of training per week (*F*_(1, 103)_ = 0.48, *n.s*.). Additionally, female and male football players differed significantly regarding their years of experience in football favoring male football players with more years of experience (*F*_(1, 104)_ = 26.36, *p* < 0.01). Yet, no differences are present in the competition level, since all participants are playing on a state level.

The overall representative team consisted of 56 young talented football players, while the non-representative team had 49 players. There were no significant differences between the two groups in terms of age (*F*_(1, 103)_ = 1.944, *n.s*.), training frequency (*F*_(1, 103)_ = 0.011, *n.s*.), weekly training hours (*F*_(1, 103)_ = 0.024, *n.s*.), or processing speed (*F*_(1, 103)_ = 2.077, *n.s*.). Including sex as an additional factor, no differences between the representative and non-representative teams regarding age (*F*_(1, 101)_ = 1.365, *n.s*.), training frequency (*F*_(1, 101)_ = 0.875, *n.s*.), weekly training hours (*F*_(1, 101)_ = 0.698, *n.s*.), or processing speed (*F*_(1, 101)_ = 0.764, *n.s*.) existed. The absolute values are shown in Table 1.

**Table 1:** Demographical data for the participants separated for sex in total and for the representative team and the non-representative team (M, SD).

**Variable**	**Overall**		**Representative team**		**Non-representative team**	
	**Female** ***N*** = **59**	**Male** ***N*** = **47**	**Female** ***N*** = **27**	**Male** ***N*** = **30**	**Female** ***N*** = **32**	**Male** ***N*** = **17**
Age	12.31 ± 0.57	12.61 ± 0.49	12.41 ± 0.50	12.60 ± 0.50	12.22 ± 0.61	12.65 ± 0.49
Training frequency	3.84 ± 0.89	3.91 ± 1.21	3.74 ± 0.81	3.98 ± 1.40	3.94 ± 0.95	3.79 ± 0.79
Weekly training hours	5.78 ± 1.60	6.04 ± 2.26	5.86 ± 1.34	5.98 ± 2.38	5.72 ± 1.78	6.15 ± 2.08
Years of football	6.90 ± 1.60	8.38 ± 1.31	7.20 ± 1.50	8.53 ± 1.14	6.64 ± 1.65	8.12 ± 1.58
Processing speed	119.74 ± 11.59	109.57 ± 12.98	119.08 ± 12.61	108.60 ± 11.81	120.28 ± 10.86	111.29 ± 15.06

### Sex differences

First, the results regarding sex differences within the total sample are presented. Due to the significant difference in processing speed between sex, the variable processing speed has been considered as a covariate in all the following analysis.

No sex differences were observed in reaction time or accuracy rate for the three conditions of the inhibition task (congruent, incongruent and neutral), as shown in Table 2.

**Table 2:** Results of the Flanker test, differentiated between congruent, incongruent and neutral trials for each comparison.

**Condition**	**Female-male**
Congruent reaction time	*F*_(1, 94)_ = 3.181, *p* = 0.078, *η^2^* = 0.033
Accuracy rate	*F*_(1, 94)_ = 0.342, *p* = 0.560, *η^2^* = 0.004
Incongruent reaction time	*F*_(1, 94)_ = 1.675, *p* = 0.199, *η^2^* = 0.018
Accuracy rate	*F*_(1, 94)_ = 0.014, *p* = 0.906, *η^2^* = 0.000
Neutral reaction time	*F*_(1, 94)_ = 2.552, *p* = 0.144, *η^2^* = 0.026
Accuracy rate	*F*_(1, 94)_ = 0.329, *p* = 0.568, *η^2^* = 0.003

**Figure 2:**
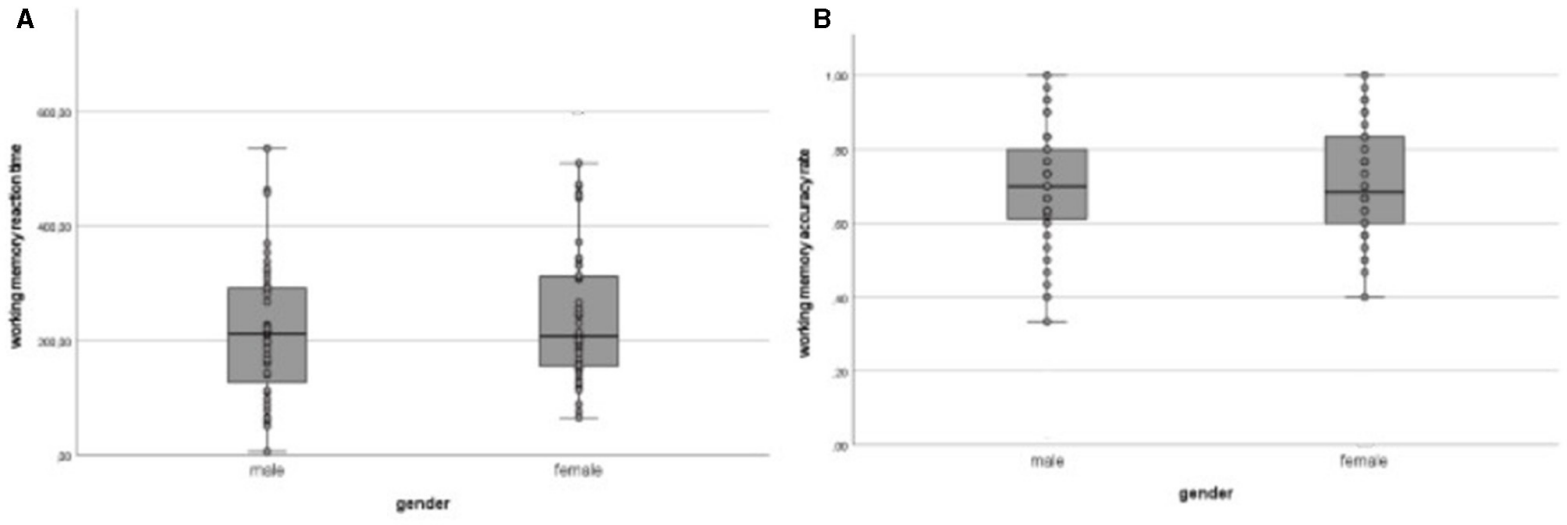
Sex specific results for the working memory task n-back. In this figure, the overall results of the n-back task of the working memory test are presented, broken down by sex. **(A)** shows the results for the average reaction time, while **(B)** displays the accuracy rate.

There were significant differences for the target reaction time in the working memory task for sex (*F*_(1, 94)_ = 4.765, *p* = *0.032*, η^2^ = 0.048), showing faster reaction times for males, while no significant differences were found for accuracy rate (*F*_(1, 94)_ = 0.000, *p* =.000, η^2^ = 0.000), as presented in Figure 2.

Additionally, no significant differences were observed for shifting costs (*F*_(1, 94)_ = 0.634, *p* = 428, η^2^= 0.007).

### Representative team and non-representative team

Below, the results regarding the selection into the representative team are presented, both overall and sex-specific.

The reaction time and accuracy rate for the three conditions of the inhibition task (congruent, incongruent, and neutral) did not differ between the two groups, as shown in Table 3. This accounts as well for the sex-specific results.

**Table 3:** Results of the Flanker test, differentiated between congruent, incongruent and neutral trials for each comparison for representative team and non-representative team overall and sex-specific.

**Condition**	**Overall: representative team – non-representative team**	**Sex-specific: representative team- non-representative team**
Congruent reaction time	*F*_(1, 96)_ = 0.839, *p* = 0.362, *η^2^* = 0.009	*F*_(1, 94)_ = 2.655, *p* = 0.107, *η^2^* = 0.027
Accuracy rate	*F*_(1, 96)_ = 0.276, *p* = 0.601, *η^2^* = 0.003	*F*_(1, 94)_ = 0.000, *p* = 0.985, *η^2^* = 0.000
Incongruent reaction time	*F*_(1, 96)_ = 0.025, *p* = 0.875, *η^2^* = 0.000	*F*_(1, 94)_ = 2.068, *p* = 0.154, *η^2^* = 0.022
Accuracy rate	*F*_(1, 96)_ = 0.399, *p* = 0.529, *η^2^* = 0.004	*F*_(1, 94)_ = 1.104, *p* = 0.296, *η^2^* = 0.012
Neutral reaction time	*F*_(1, 96)_ = 0.008, *p* = 0.929, *η^2^* = 0.000	*F*_(1, 94)_ = 0.649, *p* = 0.423, *η^2^* = 0.007
Accuracy rate	*F*_(1, 96)_ =1.850, *p* = 0.177, *η^2^* = 0.019	*F*_(1, 94)_ = 1.077, *p* = 0.302, *η^2^* = 0.011

When analyzing the target reaction time and accuracy rate in the working memory task, no differences between representative team and non-representative team overall (RT: *F*_(1, 96)_ = 1.298, *p* = 0.257, η^2^= 0.013; ACC: *F*_(1, 96)_ = 1.980, *p* =.163, η^2^ = 0.020) nor sex-specific were found (RT: *F*_(1, 94)_ = 0.632, *p* = 0.429, η^2^= 0.007; ACC: *F*_(1, 94)_ = 0.001, *p* = 0.976, η^2^ = 0.000).

Furthermore, no significant differences were found for the shifting costs between the two groups overall (*F*_(1, 96)_ = 1.471, *p* = 0.228, η^2^ = 0.015) nor sex-specific (*F*_(1, 94)_ = 0.004, *p* = 0.952, η^2^ = 0.000)).

An overview of all data, separated for sex and representative team, is presented in Table 4.

**Table 4:** Descriptive data overall and separated for the representative team and the non-representative team (M, SD).

**Measurement**	**Female**			**Male**		
	**Overall**	**Rep. team**	**Non-rep. team**	**Overall**	**Rep. team**	**Non-rep. team**
Resilience (total sum)	64.29 (5.29)	65.40 (5.50)	63.37 (5.03)	61.70 (9.92)	62.30 (10.28)	60.69 (9.53)
Flanker Con (RT)	877.43 (453.32)	877.53 (367.41)	869.01 (523.75)	774.24 (166.19)	766.27 (179.60)	787.68 (145.32)
Flanker Con (AR)	94.48 (9.05)	97.10 (3.33)	92.19 (11.39)	88.66 (21.46)	90.21 (18.94)	85.74 (25.95)
Flanker Incon (RT)	854.74 (310.92)	815.79 (199.03)	897.24 (379.28)	820.89 (229.73)	835.95 (247.10)	795.47 (202.10)
Flanker Incon (AC)	92.71 (9.67)	94.38 (4.69)	91.05 (12.24)	89.97 (16.40)	88.31 (19.49)	92.78 (9.04)5
Flanker Neu (RT)	851.80 (430.51)	775.87 (189.68)	921.85 (548.01)	774.15 (190.44)	774.99 (194.77)	772.74 (189.19)
Flanker Neu (AR)	94.85 (7.73)	96.13 (4.62)	93.34 (9.70)	90.63 (17.33)	90.05 (19.32)	91.61 (13.87)
Shifting Cost (RT)	393.03 (221.15)	355.00 (238.61)	418.85 (203.46)	348.67 (369.75)	322.12 (369.08)	392.49 (378.53)
WM (RT)	251.03 (131.12)	226.84 (115.53)	267.92 (141.10)	212.18 (118.82)	212.38 (115.52)	211.83 (128.04)
WM (AR)	71.67 (14.82)	69.73 (14.17)	73.78 (15.33)	70.28 (14.39)	68.85 (13.41)	72.71 (16.07)

RT, reaction time; AR, accuracy rate; WM, working memory; for the Flanker test the three different conditions (congruent, incongruent and neutral) are presented.

### Resilience

Analysis of the total resilience scale score revealed no significant differences between female and male athletes (*F*_(1, 94)_ = 2.420, *p* = 0.123, η^2^ = 0.025). Neither showed the analysis of the total resilience scale score significant differences between members of the representative team and those of the non-representative team (*F*_(1, 96)_ = 0.745, *p* = 0.390, η^2^ = 0.008) nor sex-specific (*F*_(1, 94)_ = 0.018, *p* = 0.895, η^2^ = 0.014).

## Discussion

The study results suggest that there are no differences in executive functions between female and male young talented football players. This accounts for sex differences overall as well as when including the selection for a representative team or not. The existing differences in processing speed and reaction times of working memory in relation to sex differences correspond to the differences observed in the general population, where female tend to have an advantage in processing speed and men in reaction time (Roivainen, 2011). Therefore, these differences are disregarded in this context.

Due to the female data gap in football, the study from Lehmann (2023), which detected no relationship between executive function and resilience in male young elite football players and no significant differences between players selected for a representative team and those not selected, was extended to female talented football players and to investigate differences in young talented football players. Yet, no such differences are existing in the selected sample for the present study. While (Huijgen et al. (2015), (Verburgh et al. (2014), and (Vestberg et al. (2017) showed differences in executive functions between highly talented football players and amateur football players, this study did not find such differences among young talented football players, regardless of whether they were selected for a representative team or not. This might account for the selected sample used in this study which was not as old as the sample by (Huijgen et al. (2015), nor in a talent system of the premier league as by (Verburgh et al. (2014), nor in a talent academy as by (Vestberg et al. (2017). While the previously mentioned studies demonstrated differences between highly talented football players and amateur football players, this accounts not for a sample selected on state level. While it makes sense to assess data within a club system on an admission level, this might introduce possible selection biases that could exclude potential influences at the executive function level. Therefore, it might impact the results, which are addressed in this study by including data assessment on a state level, although this is a specific soccer system in Germany. While it is clear that a possible selection has also been conducted for participating football on a state level, this might not be as selective as in a professional club system. Access to an academy offers a certain location advantage, which is not as significant in a decentralized organization and development of football players at the state level. While a broader access to football and potential talent development opportunities exists due to decentralization, an academy already implements a selection process at an earlier stage, partly due to potential capacity limitations. Therefore, the focus in the presented study was next to possible sex difference, on the inclusion of those players in the research, that are not in a football academy but rather in a state level selection process. As this was a different approach compared to other studies, even this level of the participants might either be to uniformly to detect differences in executive functions or there are no such differences in executive functions.

The results of this study are comparable with (Beavan et al. (2020), which argued that there are no differences in executive functions between football players and the general population, although this study did not include data from a general population. This suggests on the one hand reconsidering the role of executive functions in talent identification for football players and due to the non-existing differences in the present study, this assumption should be considered as one reason that the hypothesis could not be confirmed. But it also suggests on the other hand, the need of reconsidering the possible development for sport specific executive functions test since it might rather be, that these general test for assessing executive functions are not sensitive enough for detecting possible differences between a sport specific population and general. These sport-specific tests of executive functions should be tailored not only in terms of stimulus adaptation but also in the task design itself to reflect the specific demands of the sport. A valuable study that has already taken steps in this direction is the work by Klotzbier (2024), who developed a football-specific test—the Trail Dribbling Test—to assess both motor and cognitive aspects related to football-specific dribbling. This test was conducted in the age group between 14 and 17 years, which includes slightly older participants than those in the present study but still shows potential for application in this younger age group as well. Additionally, this study already included data from both male and female participants. This highlights the urgent need to further develop sport-specific testing methods, particularly for assessing cognitive abilities in a more targeted way. Expanding this area of research will allow future studies to better examine the relevance of sport-specific cognitive skills.

Together with the study by Lehmann (2023) the results indicate, that when selecting a representative team out of an already existing selection of football players, regardless of sex, physiological parameters seem to be a more reliable indicator than cognitive aspects during football gameplay. This is consistent with Scharfen and Memmert (2021), also highlighted that physiological factors, such as sprinting ability and RIEA, were crucial for success, particularly in the younger age group. These results highlighted the involvement of different factors that are important for the development of success in football players, but they show further, that a multidisciplinary approach is necessary to comprehend this interplay. These factors should encompass not only technical and tactical skills, but also coordinative abilities, in order to comprehensively capture the multifaceted demands of athletic performance. Considered collectively, (Beavan et al. (2020), Scharfen and Memmert (2021), and Lehmann (2023) and this study suggest a more prominent role of physiological parameters than cognitive aspects in talent identification in football. On the contrary, the findings of (Huertas et al. (2019) suggest that, in the context of talent development and the consideration of the relative age effect, there may be a need to place a different focus on the inclusion of cognitive abilities in talent selection. To prevent the exclusion of football talents born later in the year, including such parameters might be worthwhile. However, it is important to highlight that this might require a more specific adaptation of cognitive abilities to the challenges faced by athletes, which could facilitate their inclusion in the selection process. Additionally, it should not be overlooked that the size of the sample could have influenced these results. Yet, the complex interaction of physiological as well as cognitive aspects and their influence on performance in football is still something, that should be addressed in kind of multidisciplinary research.

While the previous research of Lehmann (2023) had included resilience in the context of executive function and young talented football players, these data were collected in the present study as well, also the main focus of the present study was the addressing of the female data gap. However, this aspect should also be briefly addressed in this discussion, as the aspect of resilience, or rather the psychological aspects, are playing an increasingly important role in the context of young elite football (e.g., Knöbel et al., 2024). While Holt and Dunn (2004) stated that in the process of becoming a successful football player resilience plays an important factor, (Secades et al. (2017) pointed out, that resilience helps to reduces stress factors. In sports, and specifically in football, resilience is often associated with experiencing lower levels of stress (Secades et al., 2017) or considered an important factor in becoming a high-level performance football player (Holt and Dunn, 2004). However, resilience is not only relevant in this context; it is also associated with greater success in talent identification and development (Sarmento et al., 2018). (Den Hartigh et al. (2024, p. 564) contextualize resilience “… as a dynamic process of bouncing back to normal functioning following stressors,” with stressors being related to, for example, load, particularly physiological load as well as cognitive load. Mecha and Rodriguez-Morales (2024) examined in their review the relation of executive functions and stress resilience, showing a positive association between those aspects represented in i.a. better working memory updating for those individuals with higher resilience levels. They argue further “…EFs can be key cognitive processes for the promotion of resilient behaviors.” (Mecha and Rodriguez-Morales, 2024, p. 18). Although these results demonstrate this relationship in an older age group, specifically from 18 years onward, this aspect also suggests that it might apply to younger children as well. The challenge will be to include specific age-related factors in the selected questionnaires and testing procedures. Even though the resilience questionnaire used in this study has been evaluated, a questionnaire more specifically adapted to the age group considered here might yield different results. While only few studies have investigated young elite football players, so far, no study has compared resilience in male and female young talented football players. Therefore, this was addressed in the current study, although no differences were found between the players. While previous research compared elite to non-elite or sub-elite players, in this study, the sample was more coherent. This might contribute to the non-existing differences in this sample. Furthermore, it could be the case, following (Ashdown et al. (2025), that in the sport-specific context, the resilient behavior of participants may be even more relevant and should be included in the analysis. In the study by (Ashdown et al. (2025), this was not solely evaluated through questionnaires, as in the present study, but also through observations. Reflecting on the lack of differences in the present study, it could be that the data collection instrument played a role. This suggests that the addressed topic should continue to be explored, but the methods used and the study design should be appropriately and critically questioned. In this regard, further research must concentrate on the development of standardized metrics that capture all facets of player performance, which then will facilitate equitable assessments that benefit teams, analysts, and fans alike. Based on the study conducted here, female athletes and young talented female athletes should be more strongly included in scientific research to close the data gap in this regard. Furthermore, additional practical implications of this study include the inclusion of psychological aspects in sports in general and in young elite sports in particular. Alongside physiological characteristics, psychological aspects must also be considered in this area due to the changing society and the accompanying psychological demands.

## Limitation

The selected sample, players already selected for regional representative teams, may have had an impact on the results. Differences between those players, who were further included into the selection process compared to those that were not selected might not be not present or detectable with the tests used in this study. Therefore, further studies should include players that are not in representative teams to detect possible differences between those players or further studies should include more sport specific cognitive tests. Furthermore, the comparison between academies in terms of talent identification by elite clubs and the approach at the state level should also be considered to determine whether the results presented here are universally applicable or are influenced by the system and existing selection procedures. The non-existing sex effects in the present study should be reconsidered with regard to the sample size. Additionally, future studies should include sports performance in this context. Due to the time constraints of the assessment, the number of applied tests was limited, and therefore, sports performance could not be included in this study. It could be further argued that the level of competition in men's football is considerably higher than in women's football, which may result in a more homogeneous and overall higher playing standard among the male participants in this study. Consequently, despite both groups representing Bavarian selection teams, a true equivalence in performance level between the male and female athletes cannot necessarily be assumed. Additionally, future studies should further differentiate the age groups, as cognitive development in this context—and specifically between the ages of 12 and 13—can be of much greater significance and undergo considerable changes. This differentiation was not addressed in the present study but should be considered in future research.

## Conclusion

While only few researches comparing male and female young talented football players is conducted, this study tries to add to the female data gap. Additionally, this is one of the few studies including the psychological aspect of resilience in the examination of young talented football players. While no relationship between the two concepts was detected, regardless of sex, further research is needed in this area.

## Data Availability

The original contributions presented in the study are included in the article/supplementary material, further inquiries can be directed to the corresponding author.

## Footnotes

^1^In this text, the term football refers to the sport commonly known as soccer in American English. This choice reflects the European academic context and terminology.

## References

[B1] Ashdown B. Sarkar M. Saward C. Johnston J. (2025). Exploring the behavioral indicators of resilience in professional academy youth soccer. J. Appl. Sport Psychol. 31, 96–120. 10.1080/10413200.2024.2361701

[B2] Beavan A. Chin V. Ryan L. M. Spielmann J. Mayer J. Skorski S.. (2020). A longitudinal analysis of the executive functions in high-level soccer players. J. Sport Exercise Psychol. 42, 349–357. 10.1123/jsep.2019-0312 32711397

[B3] Beaven A. Spielmann J. Ehmann P. Mayer J. (2022). The development of executive functions in high-level female soccer players. Percept. Motor Skills 129, 1036–1052. 10.1177/00315125221096989 35521695

[B4] Carnevale D. Elferink-Gemser M. Filgueiras A. Huijgen B. Andrade C. Castellano J.. (2022). Executive Functions, physical abilities, and their relationship with tactical performance in young soccer players. Percept. Motor Skills 129, 1477–1491. 10.1177/00315125221112236 35794712

[B5] Chang E. C-. H. Chu C-. H. Karageorghis C. I. Wang C-. C. Tsai J. H-. C. Wang Y-. S.. (2017). Relationship between mode of sport training and general cognitive performance. J. Sport Health Sci. 6, 89–95. 10.1016/j.jshs.2015.07.007 30356524 PMC6188876

[B6] Den Hartigh R. J. R. Meerhoff L. R. A. Van Yperen N. W. Neumann N. D. Brauers J. J. Frencken W. G. P.. (2024). Resilience in sports: a multidisciplinary, dynamic, and personalized perspective. Int. Rev. Sport Exercise Psychol. 1, 564–586. 10.1080/1750984X.2022.2039749 38835409 PMC11147456

[B7] Diamond A. (2013). Executive functions. Annu. Rev. Psychol. 64, 135–168. 10.1146/annurev-psych-113011-143750 23020641 PMC4084861

[B8] Eriksen B. A. Eriksen C. W. (1974). Effects of noise letters upon the identification of a target letter in a nonsearch task. Percept. Psychophys. 16, 143–149. 10.3758/BF03203267

[B9] Faul F. Erdfelder E. Buchner A. (2009). Statistical power analyses using G^*^Power 3.1: tests for correlation and regression analyses. Behav. Res. Methods 41, 1149–1160. 10.3758/BRM.41.4.1149 19897823

[B10] Formenti D. Trecroci A. Duca M. Vanoni M. Ciovati M. Rossi A.. (2022). Volleyball-specific skills and cognitive functions can discriminate players of different competitive levels. J. Strength Cond. Res. 36, 813–819. 10.1519/JSC.0000000000003519 31972828

[B11] Friedman N. P. Miyake A. Young S. E. DeFries J. C. Corley R. P. Hewitt J. K.. (2008). Individual differences in executive functions are almost entirely genetic in origin. J. Exp. Psychol. Gen. 137, 201–225. 10.1037/0096-3445.137.2.201 18473654 PMC2762790

[B12] Gantois P. Caputo Ferreira M. Lima-Junior D. Makamura F. Batista R. Fonseca F. L.. (2019). Effects of mental fatigue on passing decision-making performance in professional soccer athletes. Eur. J. Sport Sci. 20, 534–543. 10.1080/17461391.2019.1656781 31424354

[B13] Habekost T. Ovesen J. (2024). Cognition in elite soccer players: a general model. Front. Psychol. 15:1477262. 10.3389/fpsyg.2024.1477262 39723399 PMC11668572

[B14] Haugan J. A. Lervold K. Kaalvik H. (2025). A scoping review of empirical research on executive functions and game intelligence in soccer. Front. Psychol. 16:1536174. 10.3389/fpsyg.2025.1536174 40230991 PMC11994698

[B15] Hölling H. Erhart M. (2007). Verhaltensauffälligkeiten bei Kindern und Jugendlichen. Bundesgesundheitsbl 50, 784–793. 10.1007/s00103-007-0241-7 17514464

[B16] Holt N. L. Dunn J. G. H. (2004). Toward a grounded theory of the psychosocial competencies and environmental conditions associated with soccer success. J. Appl. Sport Psychol. 16, 199–219. 10.1080/10413200490437949

[B17] Huertas F. Ballester R. Gines H. J. Hamidi A. K. Moratal C. (2019). Relative age effect in the sport environment. role of physical fitness and cognitive function in youth soccer players. Int. J.Environ. Res.Public Health 16:2837. 10.3390/ijerph16162837 31398910 PMC6719027

[B18] Huijgen B. C. H. Leemhuis S. Kok N. M. Verburgh L. Oosterlaan J. Elferink-Gemser M. T.. (2015). Cognitive functions in elite and sub-elite youth soccer players aged 13 to 17 years. PLoS ONE 10:e0144580. 10.1371/journal.pone.0144580 26657073 PMC4691195

[B19] Huizinga M. Dolan C. V. van der Molen M. W. (2006). Age-related change in executive function: developmental trends and a latent variable analysis. Neuropsychologia 44, 2017–2036. 10.1016/j.neuropsychologia.2006.01.010 16527316

[B20] Jacobsen J. Matthaeus L. (2014). Athletics and executive functioning: how athletic participation and sport type correlate with cognitive performance. Psychol. Sport Exercise 15, 521–527. 10.1016/j.psychsport.2014.05.005

[B21] Kida N. Oda S. Matsumura M. (2005). Intensive baseball practice improves the go/nogo reaction time, but not the simple reaction time. Cognit. Brain Res. 22, 257–264. 10.1016/j.cogbrainres.2004.09.003 15653298

[B22] Kirchner W. K. (1958). Age differences in short-term retention of rapidly changing information. J. Exp. Psychol. 55, 352–358. 10.1037/h0043688 13539317

[B23] Klotzbier T. J. (2024). Skillful and strategic navigation in soccer - a motor-cognitive dual-task approach for the evaluation of a dribbling task under different cognitive load conditions. Front. Psychol. 15:1356892. 10.3389/fpsyg.2024.1356892 38933580 PMC11205518

[B24] Knöbel S. Weinberg H. Heilmann F. (2024). The interaction between acute emotional states and executive functions in youth elite soccer players. Front. Psychol. 15:1348079. 10.3389/fpsyg.2024.1348079 38590336 PMC10999690

[B25] Lehmann J. (2023). Is there a relationship between executive functions and resilience in youth elite soccer players? Brain Behav. 13:e3122. 10.1002/brb3.3122 37626476 PMC10570473

[B26] Lovecchio N. Manes G. Filipas L. Giuriato M. Torre A. Iaia F. M.. (2021). Screening youth soccer players by means of cognitive function and agility testing. Percept. Motor Skills 128, 2710–2724. 10.1177/00315125211040283 34404294

[B27] Luciana M. Conklin H. M. Hooper C. J. Yarger R. S. (2005). The development of nonverbal working memory and executive control processes in adolescents. Child Dev. 76, 697–712. 10.1111/j.1467-8624.2005.00872.x 15892787

[B28] Lundgren T. Högman L. Näslund M. Parling T. (2016). Preliminary investigation of executive functions in elite ice hockey players. J. Clin. Sport Psychol. 10, 324–335. 10.1123/jcsp.2015-0030

[B29] Maley J. H. Brewster I. Mayoral I. Siruchova R. Adams S. McGraw K. A.. (2016). Resiience in survivors of critical illness in the context of the survivors'experience and recovery. Ann. Am. Thorac. Soc. 13, 1351–1360. 10.1513/AnnalsATS.201511-782OC 27159794 PMC5021076

[B30] Mann D. L. Williams M. Ward P. Janelle C. (2007). Perceptual cognitive expertise in sport: a meta-analysis. J. Sport Exercise Psychol. 29, 457–478. 10.1123/jsep.29.4.457 17968048

[B31] Masten A. S. (2001). Ordinary magic. Resilience processes in development. Am. Psychol. 56, 227–238. 10.1037/0003-066X.56.3.227 11315249

[B32] Masten A. S. (2014). Global perspectives on resilience in children and youth. Child Dev. 85, 6–20. 10.1111/cdev.12205 24341286

[B33] Mecha P. Rodriguez-Morales M. (2024). Components of hot and cold executive functions and their relations to different forms of stress resilience: a systematic review. Stress Health 40:e3439. 10.1002/smi.3439 38943558

[B34] Moreira A. Aoki M. Franchini E. Machado D. Paludo A. Okano A.. (2018). Mental fatigue impais technical performance and alters neuroendocrine and automatic responses in elite young basketball players. Physiol. Behav. 196, 112–118. 10.1016/j.physbeh.2018.08.015 30172721

[B35] Myiake A. Friedman N. P. Emerson M. J. Witzki A. H. Howerter A. Wager T. D.. (2000). The unity and diversity of executive funtions and their contribution to complex “frontal lobe” tasks: a latent variable analysis. Cognit. Psychol. 41, 49–100. 10.1006/cogp.1999.0734 10945922

[B36] Oswald W. (2016). Zahlen-Verbindungs-Test ZVT (Number-Connection-Test), 3 Edn. Göttingen: Hogrefe.

[B37] Parsons S. Kruijt A-. W. Fox E. (2016). A cognitive model of psychological resilience. Exp. Psychopathol. 7, 296–310. 10.5127/jep.053415

[B38] Reitan R. M. (1956). Trail Making Test. Manual for Administration, Scoring, and Interpretation. Indianapolis: Indiana University Press.

[B39] Rogers R. D. Monsell S. (1995). Costs of a predictable switch between simple cognitive tasks. J. Exp. Psychol. Gen. 124, 207–231. 10.1037/0096-3445.124.2.207

[B40] Roivainen E. (2011). Gender differences in processing speed: a review of recent research. Learn. Individual Differ. 21, 145–149. 10.1016/j.lindif.2010.11.021

[B41] Sakamoto S. Takeuchi H. Ihara N. Ligao B. Suzukawa K. (2018). Possible requirement of executive functions for high performance in soccer. PLoS One 13:e0201871. 10.1371/journal.pone.0201871 30133483 PMC6104941

[B42] Sarmento H. Anguera M. T. Pereira A. Araújo D. (2018). Talent identification and development in male football: a systematic review. Sports Med. 48, 907–931. 10.1007/s40279-017-0851-7 29299878

[B43] Scharfen H.-E. Memmert D. (2019). Measurement of cognitive functions in experts and elite athletes: a meta-analytic review. Appl. Cognit. Psychol. 33, 843–860. 10.1002/acp.3526

[B44] Scharfen H-. E. Memmert D. (2021). Fundamental relationships of executive functions and physiological abilities with game intelligence, game time and injuries in elite soccer players. Appl. Cognit. Psychol. 35, 1535–1546. 10.1002/acp.3886

[B45] Schumacher J. Leppert K. Gunzelmann T. Strauß B. Brähler E. (2005). Die Resilienzskala - ein Fragebogen zur Erfassung der psychischen Widerstandsfähigkeit als Personenmerkmal. Zeitschrift für Klinische Psychologie und Psychiatrische Psychotherapie 53, 16–39.

[B46] Secades G. X. Molinero O. Ruiz Barquin R. Salguero A. La Vega D. e. R. y Marquez S. (2017). Resilience and recovery-stress in competitive athletes. Cuadernos de Psicologia del Deporte 17, 73–80.

[B47] Shariati A. Nasiri F. (2024). Comparison of executive functions in people with high and low resilience. J. Psychiatric Res. 179, 238–243. 10.1016/j.jpsychires.2024.09.016 39321522

[B48] Shields G. S. Moons W. G. (2017). Better executive function under stress mitigates the effects of recent life stress exposure on health in young adults. Stress 20, 75–85. 10.1080/10253890.2017.1286322 28114849 PMC5517019

[B49] Spielmann J. Beavan A. Mayer J. (2023). The relationship of personality and executive functions in high-level soccer athletes: expertise- and gender-specific differences. Front. Sports Active Living 5:1130759. 10.3389/fspor.2023.1130759 37188070 PMC10175618

[B50] Verburgh L. Scherder E. J. van Lange P. A. M. Oosterlaan J. (2014). Executive functioning in highly talented soccer players. PLoS ONE 9:e91254. 10.1371/journal.pone.0091254 24632735 PMC3954684

[B51] Vernon P. A. (1993). Der Zahlen-Verbindungstest and other trail-making correlates of general intelligence. Pers. Individual Differ. 14, 35–40. 10.1016/0191-8869(93)90172-Y

[B52] Vestberg T. Gustafson R. Maurex L. Ingvar M. Petrovic P. (2012). Executive functions predict the success of top-soccer players. PLoS ONE 7:e34731. 10.1371/journal.pone.0034731 22496850 PMC3319604

[B53] Vestberg T. Reinebo G. Maurex L. Ingvar M. (2017). Core executive functions are associated with success in young elite soccer players. PLoS ONE 12:e0170846. 10.1371/journal.pone.0170845 28178738 PMC5298906

[B54] Voss M. W. Kramer A. F. Basak C. Prakash R. S. Roberts B. (2010). Are expert athletes' 'expert' in the cognitive laboratory? A meta-analytic review of cognition and sport expertise. Appl. Cognit. Psychol. 24, 812–826. 10.1002/acp.1588

[B55] Wagnild G. M. Young H. M. (1993). Development and psychometric evaluation of the resilience scale. J. Nurs. Meas. 1, 165–178. 7850498

[B56] Zhang Y. Zhang X. Zhang L. (2019). Executive function and resilience as mediators of adolescents' perceived stressful life events and school adjustment. Front. Psychol. 10:446. 10.3389/fpsyg.2019.00446 30873099 PMC6403185

